# Identifying the participant characteristics that predict recruitment and retention of participants to randomised controlled trials involving children: a systematic review

**DOI:** 10.1186/s13063-016-1415-0

**Published:** 2016-06-22

**Authors:** Louise Robinson, Pauline Adair, Margaret Coffey, Rebecca Harris, Girvan Burnside

**Affiliations:** Department of Biostatistics, Institute of Translational Medicine, University of Liverpool, Block F Waterhouse Building, 1-5 Brownlow Street, Liverpool, L69 3GL UK; R&D Department, Salford Royal NHS Foundation Trust, Summerfield House, Stott Lane, Salford, M6 8HD UK; Health Psychology and Behavioural Medicine Research Group, School of Psychological Sciences and Health, University of Strathclyde, 40 George Street, Glasgow, G1 1QE UK; School of Health Sciences, University of Salford, Allerton Building, Frederick Road Campus, Salford, M6 6PU UK; Department of Health Services Research, Institute of Psychology Health and Society, University of Liverpool, Waterhouse Building, Liverpool, 1-5 Brownlow Street, Liverpool, L69 3GL UK

**Keywords:** Recruitment, Retention, Randomised controlled trial

## Abstract

**Background:**

Randomised controlled trials (RCTs) are recommended as the ‘gold standard’ in evaluating health care interventions. The conduct of RCTs is often impacted by difficulties surrounding recruitment and retention of participants in both adult and child populations. Factors influencing recruitment and retention of children to RCTs can be more complex than in adults. There is little synthesised evidence of what influences participation in research involving parents and children.

**Aim:**

To identify predictors of recruitment and retention in RCTs involving children.

**Methods:**

A systematic review of RCTs was conducted to synthesise the available evidence. An electronic search strategy was applied to four databases and restricted to English language publications. Quantitative studies reporting participant predictors of recruitment and retention in RCTs involving children aged 0–12 were identified. Data was extracted and synthesised narratively. Quality assessment of articles was conducted using a structured tool developed from two existing quality evaluation checklists.

**Results:**

Twenty-eight studies were included in the review. Of the 154 participant factors reported, 66 were found to be significant predictors of recruitment and retention in at least one study. These were classified as parent, child, family and neighbourhood characteristics. Parent characteristics (e.g. ethnicity, age, education, socioeconomic status (SES)) were the most commonly reported predictors of participation for both recruitment and retention. Being young, less educated, of an ethnic minority and having low SES appear to be barriers to participation in RCTs although there was little agreement between studies. When analysed according to setting and severity of the child’s illness there appeared to be little variation between groups. The quality of the studies varied. Articles adhered well to reporting guidelines around provision of a scientific rationale for the study and background information as well as displaying good internal consistency of results. However, few studies discussed the external validity of the results or provided recommendations for future research.

**Conclusion:**

Parent characteristics may predict participation of children and their families to RCTs; however, there was a lack of consensus. Whilst sociodemographic variables may be useful in identifying which groups are least likely to participate they do not provide insight into the processes and barriers to participation for children and families. Further studies that explore variables that can be influenced are warranted. Reporting of studies in this field need greater clarity as well as agreed definitions of what is meant by retention.

**Electronic supplementary material:**

The online version of this article (doi:10.1186/s13063-016-1415-0) contains supplementary material, which is available to authorized users.

## Background

Randomised controlled trials (RCTs) are generally recognised as the ‘gold standard’ in evaluating the effectiveness of health care interventions [1]. However, the reliability of results can be compromised when non-random subsets of participants who enrol or remain on a study are significantly different from those who choose not to take part or subsequently drop out [2].

Difficulties surrounding the recruitment and retention of participants in RCTs are well documented [3], and many clinical trials are stopped or extended due to issues surrounding recruitment and retention [4]. A review of RCTs based on recruitment methods carried out in 2006 reported that up to 60 % of RCTs either fail to meet their recruitment targets or request extensions due to delayed recruitment [5]. Similarly, reviews of UK-based trials have found that less than 31 % of publicly funded trials in the UK achieved their original recruitment target between 2002 and 2008 [3].

Previous studies have suggested that a greater understanding of who is more likely to decline trial participation could help to identify factors that are amenable to change and provide solutions for improving recruitment and retention [6]. Furthermore, findings from studies that successfully predict which participants are less likely to participate could also be used to develop screening tools enabling researchers to provide additional support to target populations [7].

Whilst participation in RCTs in adult populations is known to be influenced by the characteristics and beliefs of the participant and their families, in child-focussed studies where the child is old enough to assent to their participation, more complex factors are involved. Parent, family *and* child characteristics can be important in determining whether the family choose to participate. Decision-making on behalf of a child is recognised to be a different experience to the adult making a decision for themselves [8, 9]. Thus, trials involving children and families can potentially have a greater number of complexities influencing recruitment and retention than adult populations [7]. Research into the reasons for participation and non-participation in child-focussed RCTs therefore warrants investigation separate to adult populations. Despite this, the majority of studies into recruitment and retention in RCTs are focussed on adult populations [10, 11].

It is commonly accepted that ethnic minority, lower socioeconomic status (SES), low income or poorly educated groups are less likely to take part in research and are, therefore, traditionally underrepresented [12–15]. These assumptions appear to be based on common findings from the analysis of single trial datasets. The literature suggests that whilst many individual studies have analysed data on participants who chose to participate against those who did not from within their own sample, very few studies have synthesised data from a range of trial datasets.

A previous systematic review of predictors to participation in cancer clinical trials, found that older age, lower SES and ethnic minority status most commonly predicted non-participation in the 65 studies included [16]. This review included four articles on adolescents or children, all finding that parental influence was an important factor. There is, however, a lack of evidence synthesis in this area regarding a wider range of types of clinical trials. The main aim of this systematic review was to identify the predictors of recruitment and retention in a range of types of RCTs involving children.

This review will be reported in line with the PRISMA guidelines for the reporting of systematic reviews [17] (see Additional file 1).

## Methods

### Search strategy and data extraction

An electronic search was carried out in MEDLINE, PsychINFO, CINAHL and the Cochrane Library (see Additional file 2). Citation searching of all ‘included’ and ‘unclear’ papers put forward after the title and abstract screening phase was conducted using the Web of Knowledge. In addition the reference section of each of the aforementioned papers was searched for further papers to include in the review. One reviewer (LR) screened titles and abstracts of all retrieved articles against the inclusion and exclusion criteria. Articles that were classified as ‘include’ or ‘unclear’ were carried forward to the next stage of screening where full-text papers were obtained. If it was evident that papers did not meet the inclusion criteria they were classified as ‘exclude’ and full-text articles were not obtained. Any uncertainties were classified as ‘unclear’ to avoid bias due to one author screening at this stage. Full-text screening was conducted by LR against the inclusion and exclusion criteria. ‘Unclear’ papers were independently reviewed by PA after the full-text screening phase. Data extraction was undertaken independently by two reviewers (LR and PA). Due to the diversity of studies and outcomes included in the articles within this review, a traditional quality assessment tool was difficult to adapt to the assessment of studies; therefore, a tool was specifically developed for this review (Additional file 2), adapted from two existing checklists [18, 19]. Each item on the 14-point checklist was scored 0–2 (0 = inadequate description, 1 = fair description, 2 = adequate description). Each paper was then given a percentage quality score (based on points attained out of total points available). The use of a 3-point rating scale was based on methods used in similar studies [16, 20].

### Study selection

Quantitative, peer-reviewed, English language studies were included if they investigated for empirical predictors of recruitment and/or retention of children to RCTs. For the purpose of this review children were defined as 0 years (birth) to 12 years (study intervention finishes before the child’s 13th birthday), this avoided possible confounding factors associated with children starting high school and, therefore, having more control over their own decision to take part [21]. For the purpose of this review, *recruitment* was defined as being randomised onto a study and, therefore, the participant had enrolled. Papers comparing participants who were randomised with those who chose not to be randomised were eligible for inclusion in the review. *Retention* was defined as a measure of whether participants remained in the study for final outcome assessment. Papers were eligible for inclusion if they had a clear definition of participants who withdrew (e.g. were withdrawn due to protocol non-compliance or chose to withdraw) and compared the characteristics of these participants to participants who remained in the study (did not withdraw or were not withdrawn from the study due to protocol non-compliance). Hypothetical trials, qualitative studies and articles without a clear definition of recruitment or retention, e.g. papers that measured engagement/participation were excluded from this review. No other exclusion criteria were applied.

Studies were categorised into *medical* (i.e. involving patients, or children who had received a diagnosis) or *non-medical* (i.e. children who were otherwise classified as healthy, including those who had been identified as ‘at risk’).

### Statistical analysis

The most frequently reported variables across the included studies were considered for meta-analysis using adjusted odds ratios of recruitment and/or retention as the outcome variables. Unfortunately, due to the heterogeneity in scales and measures it was not possible to conduct a meta-analysis on any of the sociodemographic variables identified in this review.

## Results

### Description of included studies

**Fig. 1:**
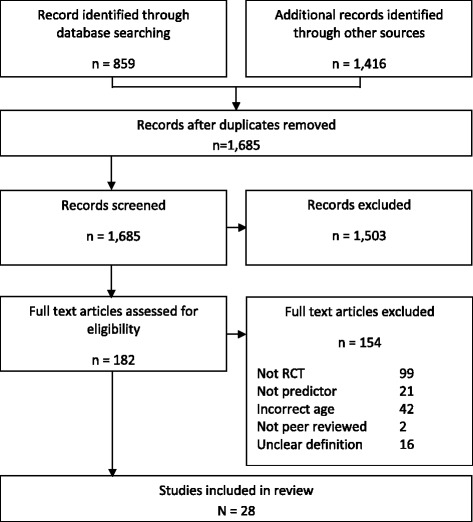
Flow diagram of phases of systematic review

A flow diagram of the screening process is presented in Fig. 1. The database search, full-paper reference and citation searches of included papers resulted in 2275 papers, 590 of which were duplicates. One thousand five hundred and three papers were excluded through screening of titles and abstracts; full-paper articles were obtained for the 75 ‘include’ and 105 ‘unclear’ for full-paper screening. The most frequent reason for exclusion after full-text screening was the study design not being an RCT and/or the intervention did not focus on children aged 0–12 years.

Twenty-eight studies met the inclusion criteria [22–49]. This gave a total of 12,504 participants being assessed for factors predictive of their participation across the 28 RCTs. Eleven studies were specifically concerned with prediction of recruitment of participants to RCTs. Eleven studies focussed on retention of participants and six studies examined predictors of both recruitment and retention to an RCT.

Of the 28 included studies, 23 RCTs were randomised at an individual level (including one crossover trial) and the remaining five studies were cluster trials. The articles reported on recruitment and retention in numerous settings including home visits, university clinics, hospitals and schools. Twelve of the studies were community-based, 11 were located in a health setting and three were carried out between community and healthcare settings (with information on setting unavailable for two studies).

The majority of articles were conducted in the US and published in 2000 or later, only four studies were published prior to this. The RCTs covered a wide range of medical conditions differing in severity from children with cystic fibrosis [36] to a nutrition-focussed prevention programme for first-time mothers [30]. Twelve studies were classified as medical in their focus, whilst the remaining 16 fell into the non-medical category. The study characteristics are summarised in Table 1.

**Table 1:** Summary of study characteristics

Author	Year	Intervention	Study Length	Study Design	Focus	Country	Sample Size	Setting	Disease Type	Medical or non medical intervention	Target Population
Aylward GP, Hatcher RP, Stripp B, Gustafson NF and Leavitt LA (1985) [22]	1985	Dexamethasone administration	Repeated visits	RCT individual	Retention	USA	645	Health setting - university centres	Prevention of respiratory distress syndrome	Medical	Babies - surviving infants
Baker CN, Arnold DH and Meagher S (2011) [23]	2011	Parenting intervention	8 weeks	RCT cluster	Recruitment	USA	106	Community - childcare centres	Parent training for preventing conduct problems	Non medical	Families of preschoolers mean age of child 4.6 years (intervention group only)
Boggs SR, Eyberg SM, Edwards DL, Rayfield A, Jacobs J, Bagner D and Hood KK (2004) [24]	2004	Parent child interaction therapy (PCIT)	Longitudinal - time unlimited, mean treatment length 13 weekly sessions	RCT individual	Retention	USA	46/61 enrolled	Unclear	Existing disruptive behaviour	Medical	Children with disruptive behaviour disorders
Byrnes HF, Miller B A, Aalborg AE and Keagy CD (2012) [25]	2012	Parenting intervention	Longitudinal but this looks at enrolment	RCT individual	Recruitment	USA	351/744 eligible	Health setting - medical centres	Substance use prevention	Non medical	Families with an 11-12 year old
Constantine WL, Haynes CW, Spiker D, Kendall-Tackett K and Constantine NA (1993) [26]	1993	3 year home visits, parent support groups and education program v normal care	3 years	RCT individual	Retention	USA	885/1302 eligible	Mixed - large urban tertiary care centres and satellite clinics for hard to reach	Low birth weight premature infants reducing health and development problems	Non medical	Babies born before 37 weeks
Cunningham CE, Boyle M, Offord D, Racine Y, Hundert J, Secord M and McDonald J (2000) [28]	2000	Parenting intervention	Enrolment	RCT cluster	Recruitment (retention not clear)	Canada	1498	Community - schools	Children at risk of disruptive behaviour disorder - parent training	Non medical	5-8 year olds with high parent reported externalising problems
Cunningham CE, Bremner R and Boyle M (1995) [27]	1995	Parenting intervention	Longitudinal	RCT individual	Retention	Canada	150	Community - community-based neighbourhood schools and community centres	Children at risk of disruptive behaviour disorder - parent training	Non medical	Junior kindergarten school children with problems at home
Damashek A, Doughty D, Ware L and Silovsky J (2011) [29]	2011	Parenting intervention	Longitudinal	RCT individual	Recruitment	USA	398	Community - home	Child maltreatment prevention	Non medical	Female caregivers with a child 1-5 years in home
Daniels LA, Wilson JL, Mallan KM, Mihrshahi S, Perry R, Nicholson JM and Magarey A [30]	2012	Parenting intervention	Longitudinal	RCT individual	Recruitment and retention	Australia	698	Community - community child health clinics	Nutrition – prevention	Non medical	1st time mothers of healthy infants
Eisner M and Meidert U (2011) [31]	2011	Parenting intervention	Longitudinal but this looks at enrolment	RCT cluster	Recruitment (retention not clear)	Switzerland	821 test group only	Community - public primary schools	Parent training (triple P)	Non medical	Children in primary school
Fernandez MA and Eyberg SM (2009) [32]	2009	PCIT	2 year follow up	RCT individual	Retention	USA	99	Health setting - PCIT Lab	Existing disruptive behaviour	Medical	3-6 year olds with Disruptive Behaviour Disorder
Firestone P and Witt JE (1982) [33]	1982	Parenting intervention	4 month programme	RCT crossover	Retention	Canada	83 families (test group only)	Health setting - psychology department hospital	Hyperactive children	Medical	Families of hyperactive children 5-9 years of age
Gross D, Julion W and Fogg L (2001) [34]	2001	Parenting intervention	1 year - 15 months	RCT cluster	Recruitment and retention	USA	155 test group only	Community - childcare centres (community bases)	Parent training	Non medical	2-3 year olds attending day care centres, serving low income families
Heinrichs N, Bertram H, Kuschel A and Hahlweg K (2005) [35]	2005	Parenting intervention	Enrolment	RCT cluster	Recruitment	Germany	186/282 enrolled, test group only	Community - schools	Prevention of emotional and behaviour problems, parent training	Non medical	3-6 year olds
Ireys HT, DeVet KA, and Chernoff R (2001) [36]	2001	Parenting intervention	15 months	RCT individual	Recruitment	USA	161	Mixed - pediatric practices and home visits	Children at risk of mental health problems because of serious ongoing physical health conditions	Medical	Mothers with children aged 7-10 months with diabetes sickle cell disease, cystic fibrosis or asthma
Katz KS, El-Mohandes PA, Johnson DM, Jarrett PM, Rose A and Cober M (2001) [37]	2001	Parenting intervention	12 months	RCT individual	Recruitment and retention	USA	286	Community - Home visits	Parenting intervention to increase use of healthcare and to increase skills in providing safe and structured child rearing	Non medical	Mothers of babies, low income
Mihrshahi S, Vukasin N, Forbes S, Wainwright C, Krause W, Ampon R, Mellis C, Marks G, Peat J (2002) [39]	2002	Parenting intervention	5 years	RCT individual	Recruitment	Australia	616	Community - home visits	Asthma – prevention	Medical	Pregnant women with asthma or father has asthma
Miller GE and Prinz RJ (2003) [40]	2003	Parenting intervention	Longitudinal	RCT individual	Retention	USA	147	Health setting - children and family centre affiliated with a university	Serious childhood aggression and conduct problems	Medical	Families with 5-9 year old boys
Moser DK, Dracup K and Doering JV (2000) [41]	2000	3 methods of cardiopulmonary resuscitation training v control	Longitudinal	RCT individual	Retention	USA	578	Unclear	Cardiac/respiratory arrest	Medical	Parents and caregivers of high risk neonates at risk of cardiac/respiratory arrest
Multicentre Otitis Media Study Group (2001) [38]	2001	Bilateral intervention tubes with and without adenoidectomy against non surgical management	12 weeks from 1st visit to randomisation	RCT individual	Recruitment	UK	1315	Health setting - 3 UK Centres - Hospitals	Otologica (hearing) Glue Ear	Medical	3y3m - 9y9m referred for otological problems (OME)
Ramos-Gomez F, Chung LH, Beristain RG, Santo W, Jue B, Weintraub J, Gansky S (2008) [42]	2008	Dental disease management	Longitudinal	RCT individual	Recruitment and retention	USA	361	Health setting - health centres	Childhood caries	Non medical	Pregnant women attending community health centres, mostly Hispanic
Roggman LA, Cook GA, Peterson CA and Raikes HH (2008) [43]	2008	Parenting intervention	Longitudinal	RCT individual	Retention	USA	564 test group only	Community - interviews by phone and home visits	Home visits for early childhood development	Non medical	Children up to age 3
Van den Akker EH, Rovers MM, Van Staaij BK, Hoes AW and Schilder AGM (2003) [44]	2003	Adenotonsillectomy	Enrolment	RCT individual	Recruitment	Netherlands	First 270 randomised children	Health setting - hospital	Adenotonsille- ctomy	Medical	2-8 years old
Vermaire JH, van Loveren C and Hoogstraten J (2011) [49]	2011	Caries prevention strategies - detail unknown	6 years	RCT individual	Recruitment	Netherlands	286	Health setting - dental practices	Caries	Non medical	6 year old in dental clinics
Wagner M, Spiker D, Inman Linn M and Hernandez F (2003) [45]	2003	Parenting intervention	Monthly home visits, look at sample up to child's first birthday	RCT individual	Retention	Canada	238	Community - home based	Behaviour	Non medical	Low income families, up to 8 months old (home visitation group only - not control)
Werba BE, Eyberg SM, Boggs SR and Algina J (2006) [46]	2006	PCIT	Longitudinal	RCT individual	Retention	USA	99	Health setting - psychology clinic in health sciences centre	Existing disruptive behaviour disorder – PCIT	Medical	Families of 3-6 year olds
Winslow EB, Bonds D, Wolchik S, Sandler I, Braver S (2009) [47]	2009	Parenting intervention	11 weeks	RCT individual	Recruitment and retention	USA	325	Mixed - home and sessions on University campus	Parenting programs for divorced mothers	Non medical	Divorced mothers with a child aged 9-12
Zebracki K, Drotar D, Kirchner H, Schluchter M, Redline S, Kercsmar C and Walders N (2003)[48]	2003	Control v session of problem solving therapy for family asthma management skills	Longitudinal	RCT individual	Recruitment and retention	USA	327	Health setting - teaching hospital	Asthma	Medical	4-12 year olds

*RCT* randomised controlled trial, *SES* socioeconomic status

### Predictor variables

A total of 155 participant factors were analysed across the 28 papers; there was considerable variation between articles in the variables that were tested for their significance to predict recruitment and retention. Most papers included an analysis of sociodemographic variables alongside treatment/condition-specific variables. Whilst the majority of studies included condition-specific (e.g. asthma severity [48], parent stress [24]) predictors of participation in their analysis, the variation in measures used was considerable, even for studies within the same field. Heterogeneity, therefore, precluded any meta-analysis.

Participant factors were classified into four categories: (1) parent characteristics, (2) child characteristics, (3) family characteristics, and (4) neighbourhood characteristics. Of the 155 variables reported, 45 parent, 19 child, 4 family and 2 neighbourhood variables were found to be significant predictors of recruitment and retention to RCTs involving children and families in at least one study. Nine parent, two child, two family and two neighbourhood characteristics were recurrent across the included papers and were analysed. The 15 recurrent predictors are presented in Table 2 (recruitment-focussed studies) and Table 3 (retention-focussed studies) and will be discussed hereon.

**Table 2:** Recruitment studies – summary of predictors

				Parent									Child		Family		Neighbourhood	
Author	Predicting	Setting	Medical or non- medical intervention	Ethnicity	Education	Parent Age	Income	SES	Parental depression	Single parenthood	Marital status	Employment	Child gender	Child age	Number of family members	Number of children	N'hood high school drop out	Density of n'hood networks
Baker et al., 2011 [23]	Recruitment	Community	Non- medical	✔				✔	X	X								
Byrnes et al., 2012 [25]	Recruitment	Health	Non- medical	✔	✔	✔							X				✔	
Constantine et al., 1993 [26]	Recruitment	Mixed	Non- medical	✔														
Cunningham et al., 2000 [28]	Recruitment	Community	Non- medical		✔		X		X	✔			✔	X				
Damashek et al., 2011 [29]	Recruitment	Community	Non- medical	X	X	X	X		✔									
Daniels et al., 2012 [30]	Recruitment	Community	Non- medical	X	✔	✔					✔							
Eisner M and Meidert U, 2011 [31]	Recruitment	Community	Non- medical	✔			✔	✔		X								✔
Heinrichs et al., 2005 [35]	Recruitment	Community	Non- medical			X		✔		✔					X			
Ireys et al., 2001 [36]	Recruitment	Mixed	Medical	✔					✔				X					
Mihrshahi et al., 2002 [39]	Recruitment	Community	Medical	X	✔	X						X						
Multi-centre Otitis Media Study Group, 2001 [38]	Recruitment	Health	Medical	X				X					X	X		X		
Van den Akkeret al., 2003 [44]	Recruitment	Health	Medical										X	X				
Vermaire et al., 2011 [49]	Recruitment	Health	Non- medical	✔				✔		X	X		X					
Winslow et al., 2009 [47]	Recruitment	Mixed	Non- medical	X	X		✔											
Zebracki et al., 2003 [48]	Recruitment	Health	Medical	X	X	✔	X				X	X			X			
			Total	12	7	6	5	5	4	5	3	2	6	3	2	1	1	1
			Significant	6	4	3	2	4	2	2	1	0	1	0	0	0	1	1
		Non- significant		6	3	3	3	1	2	3	2	2	5	3	2	1	0	0

Key: x = not significant, ✔ = significant, *SES* socioeconomic status

**Table 3:** Retention studies – summary of predictors

				Parent									Child		Family		N’hood	
Author	Predicting	Setting	Medical or non- medical intervention	Ethnicity	Education	Parent Age	Income	SES	Parental depression	Single Parenthood	Marital status	Employment	Child gender	Child age	Number of family members	Number of children	N'hood high school drop out	Density of n'hood networks
Aylward et al., 1985 [22]	Retention	Health	Medical					✔										
Boggs et al., 2004 [24]	Retention	Unclear	Medical	X		X		X					X	X				
Constantine et al., 1993 [26]	Retention	Mixed	Non- medical		✔	X												
Cunningham et al., 1995 [27]	Retention	Community	Non- medical		X				X									
Daniels et al., 2012 [30]	Retention	Community	Non- medical	X	✔	✔					X							
Fernandez MA and Eyberg SM, 2009 [32]	Retention	Health	Medical					✔										
Firestone P and Witt JE, 1982 [33]	Retention	Health	Medical		✔	✔	✔						✔	✔		X		
Gross et al., 2001 [34]	Retention	Community	Non- medical	X	X	X	X		X		✔	X	X					
Katz et al., 2001 [37]	Retention	Community	Non medical	X	X	X					X	X				✔		
Miller GE and Prinz RJ, 2003 [40]	Retention	Health	Medical		X													
Moser et al., 2000 [41]	Retention	Unclear	Medical	X	X	X	X		✔		X				X			
Ramos-Gomez et al., 2008 [42]	Retention	Health	Non- medical	✔	X	X	✔			X								
Roggman et al., 2008 [43]	Retention	Community	Non- medical		X	X			X	✔		X	✔	✔	X			
Wagner et al., 2003 [45]	Retention	Community	Non- medical		✔	✔	✔											
Werba et al., 2006 [46]	Retention	Health	Medical			✔		X	✔	X				X				
Winslow et al., 2009 [47]	Retention	Mixed	Non- medical	✔	✔		X											
Zebracki et. al., 2003 [48]	Retention	Health	Medical	X	✔	✔	X				X	X			X			
			Total	8	13	12	7	4	5	3	5	4	4	4	3	2	1	1
			Significant	2	6	5	3	2	2	1	1	0	2	2	0	1	1	1
		Non-significant		6	7	7	4	2	3	2	4	4	2	2	3	1	0	0

Key: x = not significant, ✔ = significant,

### Parent characteristics

Parent characteristics were the most common factors assessed for significance to predict recruitment and retention in RCTs; 88 parent-related predictors were included in the analyses. Nine parent characteristics were frequently assessed across the 28 studies; these were ethnicity (*n* = 17 studies), parent education (*n* = 16 studies), parent age (*n* = 16 studies), income (*n* = 10 studies), SES and parental depression (*n* = 9 studies), single parent status (*n* = 8 studies), marital status (*n* = 6 studies) and employment (*n* = 5 studies).

#### Ethnicity

Ethnicity was found to be a significant predictor of recruitment in six of the 12 studies where this variable was included. Ethnic minorities were less likely to enrol in five of the six studies that found it to be a significant predictor [23, 25, 31, 36, 49]. Constantine et al. [26] reported that ‘Blacks and Hispanics’ were more likely to enrol than ‘Whites and Others’ in their home visits for a low-birth-weight children trial based in the US. This finding, however, appears to represent confound due to the offer of free, long-term medical follow-up in a population that were less likely to have guaranteed care [26]. Six studies analysed ethnicity but did not find it to be a significant predictor. The majority of recruitment studies that found ethnicity to be a significant predictor were non-medical, only one of the six studies was in a medical intervention. Two of the studies were community-based, two were in a health-care setting and two were delivered across both settings.

Ethnicity was analysed in eight retention studies; for example, in one of the included studies Winslow et al. [47] reported that ethnic minorities were more likely to remain in their mixed setting (health- and community-based) parenting intervention for divorced mothers and Ramos-Gomez et al. [42] reported that Mexican Americans were more likely to remain on their practice-based dental prevention trial than other Hispanic or non-Hispanic populations. Six other studies found that ethnicity was not a significant predictor of retention in their samples.

#### Education

A measure of parent/caregiver education was included in seven of the recruitment trials and was found to be a significant predictor in four of these studies. Whilst the studies measured different levels of education including college [25], high school [28], university [30] and tertiary education [39] all four articles report that recruitment was predicted by higher educational attainment of parents. Two of the four studies were community-based non-medical interventions; the other two were a community-based medical and a health setting-based non-medical intervention, respectively.

Education was the most frequently examined variable in relation to retention; however, retention was only reported to be significantly impacted by higher levels of education in 6 of the 13 retention articles [26, 30, 33, 45, 47, 48]. Studies that found education to be a significant predictor of retention showed no preference for setting; however, four of the studies were non-medical interventions and two were medical.

#### Socioeconomic status

Indicators of SES varied, with no common measure being used between studies. Lower SES predicted non-participation of families in four of the five recruitment studies [23, 31, 35, 49], all of these were non-medical intervention RCTs, one being based in a health setting. Only one trial did not find SES to be a significant predictor of recruitment, this was of a medical intervention tested in a health care setting.

Two studies [22, 32] both found that low SES predicted drop out from their studies, two other studies found SES to be a non-significant predictor of retention. All four studies that reported SES were medical intervention studies, three were conducted in a health care setting, one setting was unclear.

#### Income

Some studies reported parent’s ‘income’ in the place of SES; one study [31] reported both as separate variables. Eisner and Meidert [31] found that children from dual-earner families were less likely to enrol to their trial whereas mother’s income was positively correlated with enrolment in the Winslow et al. [47] parenting intervention for divorced families. Both studies were non-medical interventions, the former was based in a health care setting with the latter being split between a health setting and the participant’s home. Three trials found that income had no impact on enrolment.

Similarly, three retention studies that investigated parent income found that higher household income parents were more likely to remain participants on their RCTs; however, a further four studies found that this was not a significant predictor of retention. There appeared to be no relationship between significance of income and setting or intervention type.

#### Age

Six studies analysed the impact of parent age on recruitment; three of the studies found that older parents were more likely to enrol. Three studies (all community-based) concluded that parent age had no impact on recruitment.

Twelve studies investigated parent age in relation to retention of participants; the majority found this to be a non-significant predictor of drop out; however, in the five studies that reported age as significant predictor, older parents were more likely to remain on the trial; these studies showed no predilection to setting or health status.

#### Other parent characteristics

Parental depression was investigated in relation to recruitment in four studies, with two finding that higher levels of depression correlated with an increased likelihood of enrolment; whereas two studies found that depression had no impact on recruitment rates. Five studies analysed parental depression in relation to retention, Moser et al. [41] concluded that parents with higher levels of depression were more likely to drop out of their trial regarding infants at risk of cardiopulmonary arrest; similarly, parents who showed higher levels of depression were more likely to withdraw from a trial delivering parent-child interaction therapy [46]. However, a further three studies found no relationship between depression and retention.

The impact of being a single parent was investigated in relation to recruitment in two parent training intervention trials; whilst Cunningham et al. [28] found that single parents were less likely to enrol, Heinrichs et al. [35] reported that it increased the likelihood of enrolment. Three studies found no impact on recruitment. Only one [43] of the three studies that measured retention of participants found that single mothers were more likely to drop out of the research.

One study into recruitment found that mothers who were married were more likely to enrol in a community-based, infant-feeding intervention trial, but that marital status had no impact on retention of their participants [30]. Similarly, one retention-focussed study found that parents in partnered relationships were significantly more likely to drop out of the prevention programme trial than parents who were married, single or foster parents [34]. Conversely, two non-community-based recruitment studies and three retention studies found marital status to have no impact on retention.

The final predictor commonly tested across studies was parent employment. Employment status was examined in two recruitment and four retention studies but was not found to be a significant predictor on the recruitment or retention of the RCT participants.

### Child characteristics

Child characteristics were less frequently reported for significance than their parents’; 56 variables were analysed across the studies; however, the majority of these variables were condition-specific and, therefore, found only in a small number of studies. The two most frequently tested variables were child age (*n* = 7 studies) and child gender (*n* = 10 studies).

#### Child age

Age of the child was examined in three recruitment studies but found to have no impact on rates of enrolment. Younger children were significantly more likely to drop out of the sample of 5–9 year-old children enrolled onto a behavioural parent-training programme [33]; the same was true in a sample of children and parents enrolled onto a home visit programme [43]. However, child age had no impact on retention of participants in two other studies in the review.

#### Child gender

Parents of boys were more likely to enrol onto parenting courses in one study [28] but had no impact on recruitment in the other studies that analysed the variable. Firestone and Witt [33] found that girls were more likely to withdraw from their hospital-based trial with hyperactive children, whereas Roggman et al. [43] found that boys were more likely to drop out of their home visit programme early. Two trials found that child gender had no impact on retention of participants.

### Family characteristics

Analysis of family variables was also less common; the two commonly assessed factors were number of children in the family/home (*n* = 3 studies) and number of people in the family (*n* = 4 studies). Only one study that investigated characteristics of the family found an impact: Katz et al. [37] found that mothers with more children were more likely to drop out than mothers with fewer children.

### Neighbourhood characteristics

Whilst identified as a separate category, neighbourhood factors were only investigated in two of the included studies. Eisner and Meidert [31] found that a greater density of neighbourhood networks predicted recruitment; however, theirs was the only study to investigate this variable. Similarly, neighbourhood high school drop out was a significant predictor of recruitment in the one study that analysed it.

### Quality assessment

Results of the quality assessment of the 28 studies are presented in Table 4. The quality of papers ranged from 89 to 46 %. Whilst the majority of papers gave a detailed background and scientific rationale, fewer papers outlined clear objectives and hypotheses for the research (*n* = 11 included a hypothesis).

**Table 4:** Quality assessment of articles (adapted from Durant [19] and von Elm et al. [18])

Authors and date	Quality assessment item (see key below)														
	1	2	3	4	5	6	7	8	9	10	11	12	13	14	%
Aylward et al.,1985 [22]	2	1	1	1	0	2	1	1	2	2	1	0	0	0	50 %
Baker et al., 2011 [23]	2	2	2	1	2	1	1	1	1	1	0	2	0	2	64 %
Boggs et al., 2004 [24]	2	2	2	2	1	1	2	2	2	0	2	2	0	1	75 %
Byrnes et al., 2012 [25]	2	2	1	1	2	1	Na	2	2	1	2	2	0	2	77 %
Constantine et al., 1993 [26]	2	1	2	2	1	2	2	1	2	1	1	0	2	0	68 %
Cunningham et al., 2000 [28]	2	1	1	1	2	1	Na	1	1	1	1	0	0	0	46 %
Cunningham et al., 1995 [27]	2	1	1	1	2	2	1	1	1	1	1	2	0	0	57 %
Damashek et al., 2011 [29]	2	2	1	1	1	1	Na	2	2	2	2	2	0	0	69 %
Daniels et al., 2011 [30]	2	1	2	2	1	1	2	2	2	1	2	2	1	1	76 %
Eisner and Meidert, 2011 [31]	1	0	1	1	1	2	1	2	2	0	1	2	1	0	54 %
Fernandez and Eyberg, 2009 [32]	2	2	2	1	1	1	1	1	1	2	1	1	0	1	61 %
Firestone and Witt, 1982 [33]	1	1	2	1	1	1	1	1	2	1	1	2	0	1	57 %
Gross et al., 2001 [34]	2	1	2	2	1	2	2	1	2	0	2	2	0	1	71 %
Heinrichs et al., 2005 [35]	2	2	2	1	2	1	Na	1	1	1	0	1	2	1	65 %
Ireys et al., 2001 [36]	2	2	2	1	1	1	Na	2	2	1	0	1	1	0	62 %
Katz et al., 2001 [37]	2	1	1	2	1	1	2	1	2	0	1	0	0	1	54 %
Mihrshahi et al., 2002 [39]	2	0	1	2	2	1	0	1	1	0	1	0	0	0	42 %
Miller and Prinz, 2003 [40]	2	2	2	2	2	1	1	1	1	1	1	0	0	2	64 %
Moser et al., 2000 [41]	2	1	2	1	2	2	2	2	2	1	1	0	0	0	64 %
Multicentre Otitis Media Study Group, 2001 [38]	2	1	2	2	1	2	Na	1	2	0	2	1	2	0	69 %
Ramos-Gomez et al., 2008 [42]	2	1	2	2	1	2	2	2	2	1	2	2	2	1	86 %
Roggman et al., 2008 [43]	2	1	1	1	0	2	0	1	1	1	2	0	0	2	50 %
Van den Akker et al., 2003 [44]	1	1	1	1	1	2	Na	1	2	0	1	1	1	0	50 %
Vermaire et al., 2011 [49]	2	1	1	1	0	2	Na	2	2	2	2	0	1	1	65 %
Wagner et al., 2003 [45]	2	1	0	2	1	1	1	1	2	1	2	2	2	2	71 %
Werba et al., 2006 [46]	2	2	0	2	2	1	1	2	2	1	2	2	0	1	71 %
Winslow et al., 2009 [47]	2	2	2	1	2	1	Na	2	2	2	2	2	0	2	85 %
Zebracki et al., 2003 [48]	2	2	2	2	2	1	2	2	2	1	1	2	2	2	89 %

Key

1. Does the paper explain the scientific background and rationale for the investigation being reported?

2. Are specific objectives stated, including any pre-specified hypotheses?

3. Are key elements of study design and original trial explained in enough detail?

4. Are setting, locations, and the study sample described clearly in terms of sample size and characteristics?

5. Are lengths of exposure/intervention provided for applicable groups, i.e. control and intervention or just intervention if only measuring this group?

6. Is the study size large enough to test the hypotheses?

7. If a longitudinal retention study, are details given of the efforts to maintain the sample? i.e. payments, contacts made etc.?

8. Are the findings presented clearly, objectively and in sufficient detail to enable the reader to judge the results for himself/herself?

9. Are the findings internally consistent, i.e. do the numbers add up properly, can the different tables be reconciled, etc.?

10. Were appropriate variables or factors controlled for or blocked during the analysis?

11. Do the investigators present sufficient data in tables and in the text to adequately evaluate the results?

12. Are limitations of the study discussed, taking into account sources of potential bias or imprecision?

13. Do the authors discuss the generalisability (external validity) of the study results?

14. Are recommendations for future research made?

Score

0 – inadequate description

1 – fair description

2 – adequate description

Most papers gave sufficient detail on the trial from which data originated to understand the study design, populations and settings; however, two of the studies [45, 46] did not include sufficient detail for the reader to understand the nature of the trial. Similarly, three of the 26 studies did not detail the intervention, including length of exposure to the intervention. All of the studies were judged to have provided an objective account, with sufficient detail and explanation of the method of analysis and results for the reader to have a sound understanding and judge the results for themselves. None of the included studies raised concern regarding the internal consistency of the findings. It was felt that three of the included studies [23, 35, 36] did not present findings in clear tables. Heinrichs et al. [35] conducted logistic regression including a number of sociodemographic variables and parent/family characteristics but did not present the results. Similarly, Baker et al. [23] conducted statistical analysis including chi-square tests, *t* tests and logistic regression analysis; however, results of tests are only reported in free text and are difficult to comprehend as a consequence. In some instances it was difficult to extract results including one [26] that only reported significant predictors and did not present results for non-significant predictors; similarly, Aylward [22] did not report results of the statistical analysis for the full range of predictors. This made it difficult to compile results during data extraction as it was not clear whether predictors not reported were not statistically significant or were not included in the testing. In six of the 26 studies the authors provided no detail on whether it was necessary to control for confounding variables during analysis. In such cases, studies were scored ‘0’. Only seven of the studies gave detailed recommendations for future research, whilst only six of the 28 included studies discussed the external validity of their findings.

## Discussion

This systematic review of 28 RCTs has identified several significant predictors of recruitment and retention for children and their families. A wide range of parent, child, family and neighbourhood factors have been identified to predict recruitment and retention; of the 154 variables included in analyses, 66 were found to be significant in at least one study. Parent characteristics were the most commonly assessed characteristics. Given their involvement in the decision-making and informed consent process in this age group, this finding was to be expected.

Parental ethnicity was a commonly reported predictor of recruitment and retention in the RCTs, and supports findings from a previous review focussed on adult RCT recruitment and retention where ethnic minority groups were found to be less likely to agree to participate in trials [16]. The literature reports specific reasons for ethnic minorities being excluded from research as mistrust due to events in history [50–52], language needs or discrimination [23], suspicion of intervention providers and perceived racism and stigmatisation [47]. Efforts to address the inclusion of minority groups in RCTs is evident in US policy, where, since the introduction of the National Institute of Health Revitalisation Act in 1993, increased efforts have been employed to involve minorities in research including ethnic minority populations [16, 53]. These measures prevent unequal distributions of the risks and benefits of trial participation, whilst also ensuring that findings are relevant to underrepresented populations [16]. The findings of this review could indicate that such measures are still required for research involving families and children as ethnic minorities appear to be less likely to enrol in RCTs than non-minority ethnic groups. However, whilst ethnicity was a significant predictor in six recruitment studies, a further seven investigated ethnicity but did not find an association and it is, therefore, not possible to generalise this finding to all RCTs.

The relationship between SES and ethnicity, within both adult and child populations, is widely accepted to be closely correlated; with arguments put forward that they should no longer be seen as discrete variables because ethnicity interacts with, and is confounded by, social class or SES [54]. Most of the studies included in this review acknowledge the difficulties in separating SES and ethnicity. Whilst some identified the confounding effect of the two variables, not all studies evidenced that this was controlled for during analysis and it is, therefore, possible that there is shared variance in the predictive value of the interaction between two factors in the same study. The context of the study should also be considered when interpreting the results on the impact of ethnicity and SES on recruitment and retention. Ethnicity represents a complex issue relating to a range of particular cultural values and perspectives, which will be confounded by the country in which the RCT was conducted. Further research to identify particular groups at risk of non-participation within specific contexts would, therefore, be warranted.

Within this review four of the five recruitment studies and two of the four retention studies that investigated SES as a variable, identified lower SES as a significant predictor of participation in RCTs. Many authors outside of this review have suggested why minority SES status predicts non-participation in research studies. Explanations focus on the demands placed on families in lower SES categories and their having less time to devote to research given that they are struggling with immediate problems such as childcare and insufficient financial support [55], lack of time or family commitments [23], and fewer resources for daycare and transport [50]. Parents facing these challenges may have different priorities to families with fewer challenges and may be deterred from participating as a result. Families with higher levels of stress due to factors such as access to childcare, low income and single parent status are more at risk of lack of regular routine, interfering with participation of regular trial appointments, as was observed in the Roggman et al. [43] home visit programme. Non-participation of these groups could lead to non-representative results and recommendations for family interventions that are unsuitable for low SES groups, and strategies to facilitate participation are, therefore, required.

Parent income was analysed in ten studies within this review; however, only one of these also had a separate measure of SES [31]. SES is commonly a combined measure of income, education and occupation and the results for income and SES are, therefore, likely to be linked. In this review, higher income seemed to predict participation in some studies and, therefore, fits with the SES trend discussed above. The studies hypothesised that low-income families are more likely to face the problems linked to SES, i.e. problems with childcare, lack of transportation, less regular work schedules [47] and more challenges than affluent families [45]. In contrast, employment, commonly used in SES calculation, showed no impact on recruitment or retention in any of the five studies that analysed it.

Higher level of parental education was also found to be positively correlated with increased recruitment and retention in 11 studies. Explanations for this finding from within this review suggest that parents with less education may have a lack of interest due to non-comprehension of the goals and how research is conducted [39]. Other researchers [47] argue that higher-educated parents may value education and research more, and their occupations may allow greater flexibility and control over their work schedules to attend appointments than employed parents with lower educational attainment. Similarly, a qualitative vaccine research study found that parents’ decision-making was impacted by how much experience a parent has in science and medicine, and therefore those with experience of research through education would be more likely to take part [56]. Studies have also suggested that less educated parents may not fully understand the altruistic value of research [57] and are, therefore, less likely to take part if they do not perceive it to be relevant to them.

Evidence from the trials included in this review suggests that older parents are more likely to enrol and remain on trials with their children. The specific reason for age being a predictor of participation is less well documented than the other variables and would, therefore, warrant further investigation in future studies. The impact of being a younger parent was investigated in one study that suggested that the older parents in a behaviour study may have tried everything else and, therefore, saw more value in remaining in the research or were ‘desperate’ for help [33]. The other three retention studies that found this predictor significant provided little explanation for the finding; however, reasons could be linked to different priorities between younger and older parents or that being younger, with lower income or being a single parent is indicative of higher levels of stress and differing priorities because of this [43].

The findings on parental depression were less conclusive, with conflicting results between studies. Similarly, the impact of marital status and single parenthood were difficult to interpret due to contradictory effects and non-significant results. Despite the relative lack of involvement from children in the decision-making process at this age, child characteristics were also frequently tested for their ability to predict recruitment and retention. The majority of child variables were condition-specific clinical variables; however, age and gender were common across a range of studies and allowed some comparison. The relatively small number of studies and disagreement between studies also made it difficult to draw conclusions on the impact of these variables.

An original objective of this review was to investigate the impact of study setting and child health-status. The relatively low number of studies that analysed each variable, and the presence of non-significant findings made it difficult to draw firm conclusions on these study-level variables and would warrant further investigation in future research.

### Implications for future research

The quality assessment highlighted differences in reporting standards across studies that predict recruitment and retention of participants in RCTs. How results were reported differed across studies, with some studies excluding non-significant predictors from their results and other ambiguous exclusions making results difficult to draw conclusions from. Additionally, 17 different definitions of ‘retention’ were identified across the studies. The findings of this study highlight the need for standardised reporting for future studies that report predictors of recruitment and retention. Research in this area would benefit from agreed common predictors and standardised variables (relevant to their field), as well as clearer definitions of recruitment and retention. Standardised definitions and consistency in reporting would allow ease of comparison between studies.

This study suggests that the groups commonly identified as at risk of poor recruitment and retention in RCTs involving children are analogous with studies aimed at adults. Several recruitment and retention strategies have been identified as successful in systematic reviews; however, the focus has been on adult populations [15, 58–60] or disease-specific areas of children’s research [61, 62]. Such techniques may be transferrable to child-focussed RCTs; however, research into transferability and effectiveness within specific health areas would be warranted.

### Limitations

One limitation is the wide range of studies compared. Whilst also being a strength of the review, the broad number of health topics, settings and intervention types could limit the validity of findings due to the range of possible confounding factors. Whilst effort was made to compare commonly used predictors across the studies to ensure consistency and comparability, there was variation within these due to the measures, data collection methods and analysis not being consistent across the 28 included papers. Most notably indicators and analysis of SES varied, with some studies using parent income as an indicator of SES whilst other studies treated this as a discrete variable. As addressed previously, whilst SES was controlled as a confounding variable in some analyses, this was not true in all papers. The authors recognise that SES may be confounded by other variables, for example parent’s education and income, but a discussion of the impact is outside the scope of this article.

The method used for quality assessments of the included studies is not standardised due to the lack of suitable tool availability. The STROBE checklist from which part of the tool was adapted is not recommended for use as a quality assessment tool but was deemed suitable due to the lack of an alternative.

A further drawback, which highlights a wider issue within this field, is the origin of studies, predominantly based in the US, Canada and Europe. Whilst geographical setting was not an exclusion criteria, this review did not identify any studies from lower-income countries. The validity of findings to non-Caucasian dominated populations is, therefore, confined by this limitation. Similarly, the exclusion of non-English language papers could also limit the findings of this study. However, no full-text articles were excluded for this reason and the impact is, therefore, minimal.

## Conclusion

This review found that the commonly assessed predictors of recruitment and retention can be categorised into parent characteristics, child characteristics, family characteristics and neighbourhood characteristics. The most commonly assessed variables were related to the parent. It would appear that younger, less educated parents from ethnic minorities and low SES groups are least likely to participate in RCTs; however, these variables were also found to be non-significant predictors in multiple studies in this review. There is no conclusive evidence to suggest that any one parent, child, family or neighbourhood characteristic can be used to predict recruitment or retention of children and their families to all RCTs. The predictors should, therefore, be treated with caution.

Similarly, the review has identified some predictors that are more commonly significant in different settings and health statuses; however, the presence of similar non-significant findings prevent clear conclusions from being drawn.

The common variables discussed within this review are difficult for the researcher to influence, and there is little in the way of understanding on how recruitment and retention strategies can be applied to the groups that are most at risk of non-participation, particularly as the majority of work in this field has been conducted in adults and the applicability of strategies with children and families is under explored. Further research into the actual barriers and processes would, therefore, be beneficial alongside investigation into what recruitment and retention strategies are most effective in this population. Qualitative methods could be utilised for an in-depth exploration of the barriers and facilitators with existing trial populations. Further investigation into study level variables would provide further insight into the impact of study setting and health status/intervention type on the predictors of recruitment and retention.

Reporting of studies in this field would benefit from greater clarity as well as agreed definitions of what is meant by retention.

## Abbreviations

RCT, randomised controlled trial; SES, socioeconomic status

## Additional files

Additional file 1: PRISMA checklist - recommended items to address in a systematic review. (DOC 63 kb) Additional file 2: Search terms and strategy – systematic review database search terms and search strategy. (DOCX 15 kb)
