# Supplementary material for: Identifying the participant characteristics that predict recruitment and retention of participants to randomised controlled trials involving children: a systematic review
Source: Trials. 2016 Jun 22;17:294. doi: 10.1186/s13063-016-1415-0 (PMC4918126; doi:10.1186/s13063-016-1415-0)
Supplement: Additional file 2: — Search terms and strategy – systematic review database search terms and search strategy. (DOCX 15 kb) [file 13063_2016_1415_MOESM2_ESM.docx]

**Additional File 1 – Search Terms and Strategy (adapted for each database)**

| **Medline (1946 to October week 3 2012)** | |
| --- | --- |
| 1 | ((Predict$ or influenc$ or motivat$ or measur$ or determin$ or estimate$ or differenti$ or compar$) adj5 (Recruit$ or participat$ or consent$ or Retention or attrition or Loss to follow-up or Dropout$ or withdraw or non-participation)) |
| 2 | (child$ or baby or infant or pediatric$ or paediatric$) |
| 3 | (exp Patient Dropouts or exp Patient Participation or exp Prospective Studies) |
| 4 | 1 AND 2 AND 3 |
|  |  |
| **PsychInfo (1806 to October week 3 2012)** | |
| 1 | ((Predict* or influenc* or motivat* or measur* or determin* or estimate* or differenti* or compar*) adj5 (Recruit* or participat* or consent* or Retention or attrition or Loss to follow-up or Dropout* or withdraw or non-participation)) |
| 2 | (child* or baby or infant or pediatric* or paediatric*) |
| 3 | (exp Treatment Dropouts or exp Experimental Attrition or exp Experimental Subjects) |
| 4 | 1 AND 2 AND 3 |
| **Cinahl (no date restrictions)** | |
| 1 | ((Predict* or influenc* or motivat* or measur* or determin* or estimate* or differenti* or compar*) N5 (Recruit* or participat* or consent* or Retention or attrition or Loss to follow-up or Dropout* or withdraw or non-participation)) |
| 2 | (child* or baby or infant or pediatric* or paediatric*) |
| 3 | (exp Research Subject recruitment or exp Research Dropouts or exp Prospective Study OR Patient Selection) |
| 4 | 1 AND 2 AND 3 |
|  |  |
| **Cochrane (no date restrictions)** | |
| 1 | ((Predict* or influenc* or motivat* or measur* or determin* or estimate* or differenti* or compar*) NEAR (Recruit* or participat* or consent* or Retention or attrition or Loss to follow-up or Dropout* or withdraw or non-participation)) |
| 2 | (child* or baby or infant or pediatric* or paediatric*) |
| 3 | (exp Patient dropouts or exp Patient Selection or exp Patient Compliance OR Follow Up Studies) |
| 4 | 1 AND 2 AND 3 |
